# Telemedicine of family-based treatment for adolescent anorexia nervosa: A protocol of a treatment development study

**DOI:** 10.1186/s40337-015-0063-1

**Published:** 2015-07-11

**Authors:** Kristen E. Anderson, Catherine Byrne, Alexandria Goodyear, Ryan Reichel, Daniel Le Grange

**Affiliations:** Department of Psychiatry and Behavioral Neuroscience, The University of Chicago Medicine, 5841 S. Maryland Avenue, MC 3077, Room B424, Chicago, IL 60637 USA; Department of Psychiatry, University of California, San Francisco, 3333 California Street, Laurel Heights Suite 245, Box 0503, San Francisco, CA 94143 USA; Department of Pediatrics, University of California, San Francisco, 3333 California Street, Laurel Heights Suite 245, Box 0503, San Francisco, CA 94143 USA

**Keywords:** Telemedicine, Family-based treatment, Anorexia nervosa, Adolescent medicine

## Abstract

**Background:**

Family-based treatment is an efficacious treatment available for adolescents with anorexia nervosa. Yet the implementation of this treatment, at least in the United States, is challenging due to a limited number of trained family-based treatment therapists and the concentration of these therapists in a limited number of urban centers. The use of telemedicine in the delivery of family-based treatment can increase access to this therapy for this patient population.

**Methods/Design:**

This two-year treatment development study (December 2013–November 2015) follows a two-wave iterative case series design. The study is ongoing and addresses the treatment needs of families in remote, rural, or underrepresented parts of the United States by delivering family-based treatment via telemedicine (video chat). The first six months of the study was dedicated to selecting a cloud-based secure telemedicine portal for use with participants. Recruitment for the first of two consecutive case series (*N* = 5) began during month seven. After these five patients completed treatment, a systematic review of treatment via feedback from participants and therapists related to the delivery of this model and use of technology was completed. A second wave of recruitment is underway (*N* = 5). At the end of both waves (*N* = 10), and after a second review of treatment, we should be able to establish the feasibility and acceptability of family-based treatment delivered via telemedicine for this patient population.

**Discussion:**

This study is the first attempt to deliver family-based treatment for adolescents with anorexia nervosa via telemedicine. If delivering family-based treatment in this format is feasible, it will provide access to an evidence-based treatment for families heretofore unable to participate in specialist treatment for their child’s eating disorder.

## Background

Anorexia nervosa (AN) is a serious psychiatric disorder typically diagnosed during adolescence [1]. Adolescents who are not effectively treated are more likely to suffer the physical and psychological health impacts associated with severe and enduring AN. These include, but are not limited to, effects on growth, bone health, and cognitive, cardiac, and gastrointestinal function [2]. In addition, individuals with AN have nearly a 12-fold greater risk of death from all causes and a 57-fold greater risk from death from suicide relative to same-aged peers [3–5]. To minimize these risks, it is essential that innovative treatments are developed to effectively and efficiently treat AN as early as possible in the course of the disorder [6]. Family-based treatment (FBT) is probably the most efficacious treatment currently available for adolescents with AN [7]. However, the implementation of FBT in the United States (US) faces several significant obstacles, such as a limited number of trained FBT therapists and the concentration of these therapists in a relatively small number of urban centers.

Based on information from an FBT training institute in the US, there is currently about one certified FBT therapist for every 2000 adolescents with AN in the US. Historically, families have traveled long distances to seek treatment and have often been unable to locate a therapist and treatment team close to home. The lack of trained FBT therapists, availability of treatment, and expenses associated with travel to urban centers all contribute to limited access to specialist treatment for many families with an adolescent diagnosed with AN. Thus, the use of telemedicine to deliver FBT offers a promising option for patients and their families.

The use of telemedicine in psychiatry was first reported in 1972 [8] for both educational purposes and the treatment of patients. Early work by this group demonstrated the use of closed-circuit television could be an asset to education, training, and the treatment of psychiatric patients. Since this time, researchers have continued to utilize telephone therapy and videoconference therapy to deliver psychotherapy. For a review of the limited literature related to telemedicine and mental health, see Bakke et al. [9] and Myers et al. [10].

Bauer and Moessner [11] reviewed studies related to technology and eating disorders and suggest the use of technology-enhanced interventions can offer a potential for improved care for eating disorders. However, the use of telemedicine in the delivery of eating disorders treatment is limited to only a few studies. A case series conducted by Simpson et al. [12] found video-based cognitive-behavior therapy to be a favorable option for adults with bulimia nervosa (BN) and binge eating disorder. A larger study of adults with BN, conducted by Mitchell and his colleagues [13], concluded that telemedicine therapy had equivalent outcomes to treatment delivered in traditional face-to-face therapy. Telemedicine approaches for the treatment of AN are more limited. A case report by Goldfield and Boachie [14] provides encouraging results for the use of telemedicine to deliver adjunctive family therapy to an adolescent with AN.

Turning specifically to telemedicine and family therapy, Backhaus et al. [15] conducted a review of videoconferencing in psychotherapy. These authors found that videoconferencing achieves similar clinical outcomes to in-person psychotherapy and high user satisfaction for individual therapy, but may be more complex in regards to conducting family therapy [15]. A review of videoconferencing in family therapy conducted by Kuulasmaa et al. [16] supports the feasibility of video appointments while noting the emphasis it places on all participants using their speech effectively in sessions. Their review stresses the importance of limiting facial expressions and gestures that will be missed over video, keeping all family members in view on the video screen to keep them engaged in discussion, and meeting with families in person at least once every six months to improve the therapeutic alliance [16]. Taken together, and notwithstanding these potential obstacles, family therapy delivered via videoconferencing might be feasible in the treatment of adolescents with AN.

Given the evidence supporting the effectiveness of in-person FBT for adolescents with AN [7, 17, 18], and the difficulties surrounding limited trained therapists in a small number of urban centers, video-based therapy might allow for an increase in access to care for such families. Therefore, we are conducting a treatment development study to address the treatment needs of families in remote, rural, or underrepresented parts of the US by delivering FBT via telemedicine (videoconference). To our knowledge, this is the first to attempt to deliver FBT to adolescents with AN via telemedicine.

### Specific aims

1. To determine the feasibility and acceptability of delivering FBT to adolescents with AN utilizing telemedicine.
2. To establish effect size to enable a large-scale study of this treatment.

## Methods

### Overall study design

**Fig. 1:**
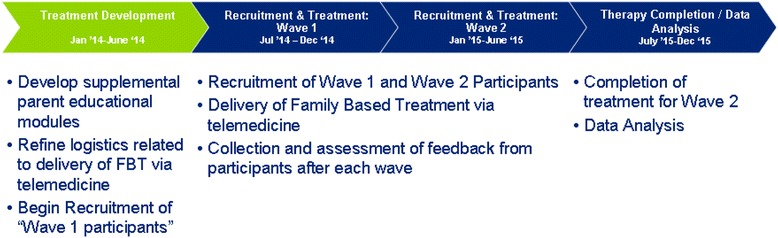
Study Timeline and Description

This is a two-year treatment development study that includes an iterative case series design – that is, two successive waves of five patients for a total sample size of 10 participants. The study started in December 2013 and will conclude in November 2015 (See Fig. 1 for the Study Design and Timeline). The first six months of the study were dedicated to locating a cloud-based telemedicine portal and completing the security and legal reviews required to utilize such a platform with research participants. Recruitment began during month 7 of Year 1, and the initial aim was to recruit 5 subjects to the first wave of this study. After this wave of participants completed treatment, a systematic review of feedback from participants and therapists related to the delivery of this model and use of technology was completed. A second wave of recruitment (Year 2: *n* = 5) is currently underway with two aims in mind: (1) to refine treatment, and (2) provide further information on the feasibility of delivering this model.

### Setting

The University of Chicago Eating Disorders Program is a specialist clinical/research service that provides outpatient treatment for adolescents with eating disorders. The multidisciplinary treatment team includes psychologists, psychiatrists, pediatricians, and clinical social workers. Following a comprehensive assessment of the eating disorder, possible comorbid psychiatric disorder(s), and medical status, outpatient treatment is provided for those families able to travel to Chicago. Treatment is typically not accessible to those families living outside a reasonable commuting distance to the city of Chicago.

### Participants

All participants will be adolescents, ages 13–18, meeting *Diagnostic and Statistical Manual of Mental Disorders*, 5th Edition [1] criteria for AN, who are at or below 87 % Expected Body Weight (EBW) and are medically stable for outpatient treatment according to the recommended thresholds of the American Academy of Pediatrics and the Society of Adolescent Medicine [19]. Patients are recruited from pediatrician offices, outpatient clinic referrals, and from other community resources. Patients and their families are provided information about the study and, if they agree to participate, complete informed consent via videoconference with the study coordinator. Full disclosure of the purpose of the study, the benefits and risks to patients’ participation, and the confidential nature of information obtained during the study is explained to participants per ethics board requirements. The Institutional Review Board of The University of Chicago, IL, and the Committee on Human Research at the University of California, San Francisco, CA, have approved this study protocol.

### Inclusion/Exclusion criteria

If a participant is on a psychotropic medication, he or she must be on a stable dose for at least 8 weeks. Also, families must have a stable Internet connection at home. Adolescents who require hospitalization for an associated physical illness, psychotic illness, or other mental illness, exhibit a current dependence on drugs or alcohol, have a physical condition (such as diabetes mellitus or pregnancy) that would influence eating or weight, or those who have completed a previous course of FBT for AN are excluded from participating in this study.

### Health portal and participant confidentiality concerns

The cloud-based health portal will allow the FBT therapist to connect with the family using a secure videoconferencing stream. The health portal also will allow families to communicate with the therapist using a messaging system (similar to E-mail). All medical information and study-related questionnaires will be managed through this program. Data security is of paramount importance to researchers. While a large number of videoconference systems are commercially available, researchers were limited to the use of Health Insurance Portability and Accountability Act (HIPAA) [20] compliant software that offered a high level of data security. As was expected, these options are far more limited than non-HIPAA compliant options. The study investigators (KEA and DLG) worked with hospital information technology staff, as well as legal services, to verify the security of the cloud-based system. Of note, this process took approximately 4 months and required systematic tests of the cloud-based system by Information Technology personnel. The cloud-based solution chosen by the current investigators complies fully with security parameters and includes stringent identity authentication, data transmission, security, privacy, and record-keeping regulations that govern the use of Protected Health Information (PHI) under the Health Insurance Portability and Accountability Act of 1996, Public Law 104–191 [20].

Secondarily, picture and sound quality are of critical importance, as the investigators want to ensure technological issues do not compromise the therapeutic experience of the patient, family, and therapist. This parameter was explored and determined to meet requirements for study implementation. Video quality will depend on the speed of the family’s Internet and the quality of their computer camera. In case the secure connection is lost, the therapist will attempt to reconnect via videoconferencing or call the family via phone.

### Intervention

#### FBT via telemedicine

FBT is an outpatient treatment that supports and encourages direct parental involvement in addressing malnutrition and other eating disorder symptoms to aid adolescents in weight restoration and recovery from the eating disorder [7, 21]. Evidence suggests that this model is particularly beneficial for adolescents with a relatively short duration of illness [7, 18, 22, 23].

For this study, treatment will be delivered per the FBT manual for adolescent AN [24], with few modifications. In addition to the fact that therapy will be delivered via videoconferencing (through a secure, HIPAA compliant, health portal), FBT will be conducted as is described in the treatment manual, i.e., all family members are required to be present for therapy. The therapist will initiate therapy sessions via videoconference and begin with an individual meeting with the patient. Following a review of the weekly weight (to be taken by parents on home scale and provided to therapist by parents through messaging system), the remaining family members (including parents, siblings, or any other relatives living in the home) will join the patient. Throughout the study, the therapist will provide guidance to the family to ensure that all family members can be seen on the video screen and that they are in a private, distraction-free location (typically a home office or family room). Therapy sessions should last 1 h for a total of 20 sessions over six months.

In Phase 1, therapy is almost entirely focused on eating disorder symptoms/behaviors and includes a family meal. Families are encouraged to strategize on how best to restore their child’s weight. Phase 2 begins when the patient is able to demonstrate compliance with parental demands and has demonstrated steady weight gain. It is at this time that the parents begin to hand control over eating back to the adolescent. Phase 3 is initiated when the patient has achieved a stable weight and self-starvation has abated. Adolescent development, unencumbered by the eating disorder, is introduced during this final phase of treatment [24].

#### Additional patient monitoring

To facilitate the appropriate treatment of medical and psychiatric comorbidities associated with AN, as well as ethically provide comprehensive treatment to this medically compromised population, study participants will continue to see their primary care physician and community psychiatrist (if applicable). The study therapist will communicate with outside physicians on a regular basis to discuss treatment progress. The online cloud-based portal will be used to facilitate communication, much like E-mail, but phone calls might be the preferred method of contact between study therapist and outside physician. It is anticipated that community psychiatrists and physicians will be open and available to working with the study therapist, and that communication will not be strained by not working in the same academic medical setting. A questionnaire to better understand the physicians’ knowledge of FBT and experience treating patients diagnosed with AN will be given to participating physicians (Wave 2).

### Measures

This study includes a standard battery of assessments conducted at baseline, mid-treatment, end-of-treatment, and six-month follow-up. Table 1 provides a summary of these assessments, which are conducted by a bachelor’s level assessor with specialized training in the administration of standardized eating disorder measures. Each assessment will be completed using the cloud based health portal and will be conducted using the videoconference feature. All questionnaires are delivered using the online health portal through a “forms feature,” which allows participants and their families to complete the questionnaires online.

**Table 1:** Assessment Battery and Schedule

Measure	Baseline	Within treatment	EOT	Assessed by
EDE	X	Session 10	X	RA
Weight (lbs)	X	Every session	X	RA/Therapist
PEDE	X	1,2,10	X	RA
MINIKID	X	1,2,10	X	RA
PEDEQ	X	1, 2, 10	X	RA
EDEQ	X	1, 2, 10	X	RA
Family Questionnaire	X	1, 2, 10	X	RA
RSE	X	1, 2, 10	X	RA
Telemedicine Feedback	X	1, 2, 10	X	RA
Physician Feedback	X		X	
BDI	X	1, 2, 10	X	RA
Parent Eval of Treatment			X	RA
Patient Eval of Treatment			X	RA

*EDE* eating disorders examination, *PEDE* parent eating disorder examination, *PEDEQ Parent Eating Disorder Examination Questionnaire, EDEQ* eating disorders examination questionnaire, *RSE* Rosenberg Self Esteem Scale, *MINIKID MINI* International Neuropsychiatric Interview for Children and Adolescents, *Telemedicine Feedback* form for families to provide feedback re: use of telemedicine; *Physician Feedback* form for physicians to provide information re: experience with FBT, *BDI* beck depression questionnaire, *Patient/Parent Eval of Treatment* qualitative interview of patient/parent experience using cloud based portal

*Eating Disorder Examination (EDE)* [25] is a standardized investigator-based interview that measures the severity of the characteristic psychopathology of eating disorders. The EDE also generates operational eating disorder diagnoses. It is a measure of present state, and, with the exception of the diagnostic items, it is exclusively concerned with the preceding four weeks. It assesses both the frequency of key behaviors (including various forms of overeating and purging) and the severity of psychopathology along certain dimensions (dietary restraint, concern about eating, concern about shape, and concern about weight). The psychometric properties of the EDE are sound and have been used in many treatment studies [26–28].

*Mini International Neuropsychiatric Interview for Children and Adolescents (MINI Kid)* [29] is a structured diagnostic interview that assesses the presence or absence of psychiatric disorders according to *DSM-IV*. The administration time is approximately 15 min and focuses on the existence of current disorders. For each disorder, one or two screening questions rule out the diagnosis when answered negatively.

*Parent Version of the Eating Disorder Examination (PEDE)* [30] is an investigator-based interview that is based on the Eating Disorder Examination. The PEDE asks for parental perspective in the recent history of their child’s eating concerns, with questions formatted similarly to the EDE. It assesses both the frequency of key behaviors (including various forms of overeating and purging) and the severity of psychopathology along certain dimensions (dietary restraint, concern about eating, concern about shape, and concern about weight).

*Eating Disorder Examination-Self-Report Questionnaire Version (EDE-Q)* [31, 32] will be used to assess restraint at baseline, within treatment, and end of treatment. The EDE-Q is a self-report measure that was adapted from the interview-based EDE. The EDE-Q measures disordered eating over a 28-day period, with some symptoms being assessed for two and three month periods as well. The EDE-Q uses the four subscales from the EDE. The EDE-Q is as reliable as it is with adults in both adolescents with AN [33] as well as those with BN [27].

*Parent Version of the Eating Disorder Examination Questionnaire (PEDE-Q)* [34] is a self-report questionnaire that measures the severity of the characteristic psychopathology of eating disorders and will be administered at baseline, within treatment, and end of treatment. This particular questionnaire asks parents/guardians to rate their child’s symptoms and is based on the EDE-Q. The PEDE-Q values the perspective of a parent or guardian, as often they have observed certain behaviors that a child will not report. There are four subscales within the PEDE-Q, including: Restraint, Eating Concern, Shape Concern, and Weight Concern.

*The Family Questionnaire (FQ)* [35] is a 20-item self-report measure of the emotional over-involvement and critical comment subscales of Expressed Emotion and will be used to compare interviewer and participant-rated Expressed Emotion. It will be administered at baseline and end of treatment.

*Rosenberg Self-Esteem Scale (RSE)* [36] is a widely used 10-item self-report of an individual’s overall self-esteem and will be administered at baseline, within treatment, and end of treatment. The RSE has been used in previous AN treatment studies and found to predict outcome.

*Beck Depression Inventory (BDI)* [37, 38] is a 21-question scale with answers rated 0–3 and has been used in numerous studies of adolescent depression, particularly in psychotherapy [39, 40]. The BDI will be administered to the patient at baseline, within treatment, and end of treatment

*Parent Evaluation of Treatment* will be used to gather information from parents/guardians regarding their opinions of the psychotherapy in which they participated. This questionnaire will be given out at the end of treatment assessment and will be used to help determine aspects of treatment that may be most or least helpful.

*Patient Evaluation of Treatment* will be used to gather information from patients regarding their opinions of the psychotherapy in which they participated. This questionnaire will be similar to the parent evaluation but adapted for the child. It will be given out at the end of treatment assessment and will be used to help determine aspects of treatment that may be most or least helpful.

*Telemedicine Feedback Form* will be used to gather feedback from patient and family members regarding the use of telemedicine for the delivery of family-based treatment. This form will be a qualitative measure that will help the research team with future development of telemedicine treatment. This measure will be given out to patients and guardians at mid-treatment assessment and end of treatment assessment.

*6-Month Follow-Up Questionnaire* will be used to gather the current height and weight of the patient 6 months from the end of treatment and will also gather information about whether the patient has participated in any outside mental health treatment since participation in the study. This form will be administered to parents/guardians 6 months from the end of treatment.

*Health Portal User Questionnaire* will be used by the Health Portal to gather feedback about the usability of this portal from a patient/user perspective. This form will be administered following the completion of treatment in the form of a *Google* document.

#### Primary goal/Outcome

Feasibility/Acceptability of FBT via Telemedicine.

#### Secondary goal/Outcome

Effect size of changes in eating disorder psychopathology, depression, and self-esteem scores.

#### Data analysis

The feasibility of delivering FBT to adolescents with AN utilizing telemedicine will be evaluated on the basis of the following measures: (1) the number of patients/families expressing interest in participation; (2) the number of patients/families enrolled in the current trial (i.e., treatment feasibility); (3) the number and percent of patients/families that completed at least 10 sessions (i.e., treatment engagement); (4) the number of patients/families completing the full course of treatment (i.e., treatment retention); (5) evaluation of the treatment by family members (i.e., family acceptability); and (6) evaluation of the treatment by patients (i.e., patient acceptability). Within-patient standardized effect size information (i.e., Cohen’s d) from baseline to end-of-treatment will be calculated for body weight, eating psychopathology (EDE global score), depression (BDI) and self-esteem (RSE). This information will be used to provide preliminary information about treatment efficacy and inform the design of future studies.

## Discussion

This will be the first treatment study to test the feasibility of a telemedicine application of FBT. If this delivery method for FBT is shown to be feasible with minimal adaptation to standard FBT, it will hold considerable promise in terms of making this evidence-based treatment available to families beyond tertiary specialist centers.

To date, the study has completed the first of two iterative case series and started the second case series. That is, 80 % of recruitment has been completed and is on track to be concluded December 2015. Participants have been recruited from eating disorder clinic referrals and from community pediatricians. Thus far, treatment has been delivered utilizing the FBT Manual [23] with no major deviations and with high rates of attendance. Additionally, given that treatment is conducted via the Internet, all sessions have begun on time and no weather, traffic, or other delays have been noted. Due to issues with eye contact on the videoconference system, the therapist utilizes patient and family members’ names regularly to identify the person being questioned and often asks parents to look at each other while discussing aspects of treatment. It should be noted that this is not unlike what occurs in face-to-face sessions. Additionally, the therapist utilizes a paper-based weight chart and shows the family the progress on the chart through the video camera.

For Wave 2 of the study, families will be able to utilize a digital weight chart that will be part of their online library to access real-time weight restoration progress. Overall, there have been no limitations identified that reduce the feasibility of implementing this model utilizing telemedicine. Some technological issues have been identified, including limited access to the system due to faulty Internet connections, difficulty in sound quality due to poor Internet connections, and use of unsupported web browsers. These issues have been remedied and have not majorly impacted implementation of treatment. Further refinement of digital documents, communication tools to connect therapist, family, and patient, as well as improvements to technology could enhance the delivery of this model.

The current study will highlight the feasibility of delivering FBT through a secure Internet portal, demonstrate the degree to which FBT delivered via telemedicine is acceptable to both families and therapists, and be able to highlight the unique aspects of FBT that require adaptation when delivered in this format. This study is important in terms of the dissemination and availability of FBT beyond tertiary treatment centers.

## Abbreviations

AN: Anorexia nervosa
BDI: Beck depression inventory
BN: Bulimia nervosa
EDE: Eating disorders examination
EDE-Q: Eating disorder examination-self-report questionnaire version
EBW: Expected body weight
FBT: Family-based treatment
FQ: Family questionnaire
HIPAA: Health insurance portability and accountability act
MINI Kid: Mini International neuropsychiatric interview for children and adolescents
PEDE: Parent version of the eating disorder examination
PEDE-Q: Parent version of the eating disorder examination questionnaire
PHI: Protected health information
RSE: Rosenberg self-esteem scale
US: United States
